# Predicting performance in attention by measuring key metabolites in the PCC with 7T MRS

**DOI:** 10.1038/s41598-024-67866-1

**Published:** 2024-07-24

**Authors:** M. Collée, R. Rajkumar, E. Farrher, J. Hagen, S. Ramkiran, G. J. Schnellbächer, N. Khudeish, N. J. Shah, T. Veselinović, I. Neuner

**Affiliations:** 1 Department of Psychiatry, Psychotherapy and Psychosomatics, RWTH Aachen University, Aachen, Germany; 2 Institute of Neuroscience and Medicine 4, INM-4, Forschungszentrum Jülich GmbH, 52425 Jülich, Germany; 3 JARA – BRAIN – Translational Medicine, Aachen, Germany; 4 Institute of Neuroscience and Medicine 11, INM-11, Forschungszentrum Jülich, Jülich, Germany; 5 Department of Neurology, RWTH Aachen University, Aachen, Germany

**Keywords:** 7T MRS, Cognition, Attention, GABA, Glutamate, Glutamine, Inositol, Aspartate, Healthy, Posterior cingulate cortex, Cognitive neuroscience, Predictive markers

## Abstract

The posterior cingulate cortex (PCC) is a key hub of the default mode network and is known to play an important role in attention. Using ultra-high field 7 Tesla magnetic resonance spectroscopy (MRS) to quantify neurometabolite concentrations, this exploratory study investigated the effect of the concentrations of *myo*-inositol (*Myo*-Ins), glutamate (Glu), glutamine (Gln), aspartate or aspartic acid (Asp) and gamma-amino-butyric acid (GABA) in the PCC on attention in forty-six healthy participants. Each participant underwent an MRS scan and cognitive testing, consisting of a trail-making test (TMT A/B) and a test of attentional performance. After a multiple regression analysis and bootstrapping for correction, the findings show that *Myo*-Ins and Asp significantly influence (*p* < 0.05) attentional tasks. On one hand, *Myo*-Ins shows it can improve the completion times of both TMT A and TMT B. On the other hand, an increase in aspartate leads to more mistakes in Go/No-go tasks and shows a trend towards enhancing reaction time in Go/No-go tasks and stability of alertness without signal. No significant (*p* > 0.05) influence of Glu, Gln and GABA was observed.

## Introduction

The posterior cingulate cortex (PCC) has been an area of research interest since the mid-2000s when it was discovered to be part of the default mode network (DMN)^1–3^. The DMN is a group of brain regions that are active while “doing nothing” or “mind-wandering” and less active when attentional networks are operating, i.e. when attention is directed towards a task^4,5^. As a key hub of the DMN, the PCC resides in the inferior medial parietal lobe. Notably, the PCC demonstrates a metabolic rate approximately 40% higher than the rest of the brain and exhibits minimal fluctuations. This characteristic not only highlights its significance but also underscores its increased vulnerability through internal or external stressors, emphasising the need for a thorough understanding of its functioning^6,7^.

The altered structure and function of the PCC in certain conditions and diseases, including Alzheimer’s disease, attention deficit hyperactivity disorder (ADHD), autism and ageing, consistently indicate its importance in cognition^2,8, 9^. The PCC is known to support the balance between internal and external attention and is directly involved in cognitive processes such as attentional focus, reaction time, arousal, executive function and memory^7,10^. Attention is fundamental to survival and daily functioning, and is, therefore, of significant research interest. The PCC modulates attention by alternating between activation and deactivation, typically deactivating during tasks that require external focus^7,11^. With ageing, the ability of the PCC to modulate this balance can falter, resulting in deactivation difficulties and subsequent cognitive impairment^12,13^. The concept of attention has been considered from numerous perspectives; in the present study, we orient ourselves with the theory presented by Peterson and Posner^14^, which divides attention into three components: orienting, alerting and executive function. In light of this, attention was measured in this study by using the subtests: “Alertness” and “Go/No-go” of the Test of Attentional Performance (TAP) and the Trail Making Test A and B (TMT A/B)^15–18^.

Given the significance of the PCC in attention, data relating to metabolite concentration in the PCC were acquired using magnetic resonance spectroscopy (MRS) on a 7T ultra-high-field (UHF) MRI machine. The ultra-high magnetic field strength has the advantage of providing a higher signal-to-noise ratio (SNR) and spectral dispersion, which improves the detection and quantification of low-concentration metabolites that would otherwise remain elusive at lower fields, e.g., gamma-aminobutyric acid (GABA) and Asp^19–21^. The metabolites selected for examination in this work primarily relate to excitation and inhibition, as these aspects are most closely associated with attention.

The metabolites that play the biggest role in brain excitation are the neurotransmitters glutamate (Glu) and aspartate (Asp)^21^. Asp is not only engaged in neurotransmission but is also used for the synthesis of other brain metabolites, such as N-acetylaspartate (NAA) and N-acetylaspartylglutamate (NAAG), which also play a role in neuropsychiatry^22^. The abundant neurotransmitter Glu, when released, is converted into glutamine (Gln) by astrocytes and vice versa, leading to the Glu/Gln cycle^23,24^. In addition to Glu and Asp, the characteristic neurotransmitter GABA was investigated for its involvement in inhibition. Inhibitory and excitatory neurotransmitters have been documented as having an influence on the deactivation of the DMN, and it is of interest to explore whether they directly impact attention^25^. *Myo*-inositol (*Myo*-Ins), a metabolite that marks glial activity and implicated in osmoregulation and inflammation, is also significant in the context of attention due to its involvement in conditions with cognitive implication^26^. Its regulation and understanding are seen as a potential agent for preventing cognitive degeneration in conditions such as Alzheimer’s disease and mild cognitive impairment^27,28^.

Given the known interplay between the metabolites *Myo*-Ins, Asp, Glu, Gln and GABA in the PCC and cognitive performance, the purpose of this exploratory study was to investigate the extent to which concentrations of these metabolites affect cognitive performance as measured by some standard neuro-cognitive tasks (TMT A/B, TAP Alertness, TAP Go/No-go) in healthy individuals, and whether concentration levels of these metabolites can be used to predict attention performance.

## Methods

### Participants

Forty-six participants, aged between 18 and 40, were included in this study (mean age: 25.4 years, SD: 4.8): 28 males (mean age: 25.6, SD: 3.97, range: 19–36) and 18 females (mean age: 25.2, SD: 6.01, range: 18–38). A population pyramid can be found on supplementary Fig. S1. The participants were recruited from the surrounding community and through online advertising. The requirements for participation were right-handedness and no history of neurological or psychiatric illnesses. In order to examine psychiatric inconspicuousness, the Mini International Neuropsychiatric Interview (MINI), German version 6.0.0, was used, and the Edinburgh Handedness Inventory (EHI) was used to assess right-handedness^29,30^. Contraindications for MRI were taken into account. Verbal and written informed consent was obtained from all participants and all methods were performed according to the recommendation of Declaration of Helsinki. The study protocol was approved by the Ethics Committee of the Medical Faculty of Aachen RWTH. All participants received financial compensation.

### MRS

MRS data acquisition was performed using a 7T Magnetom Terra scanner (Siemens Healthineers, Erlangen, Germany) equipped with a single Tx with a 32-channel Rx head coil from Nova Medical (Wilmongton, MA, USA). Structural images were acquired using the MP2RAGE pulse sequence (Marques et al. 2010) with the following protocol parameters: inversion-time 1 (INV1), TI1 = 840 ms; flip-angle, FA = 4°; INV2, TI2 = 2370 ms; FA = 5°. Other protocol parameters were echo-time, TE = 1.99 ms; repetition time, TR = 4500 ms; matrix-size, 320 × 300 × 208; voxel-size 0.75 × 0.75 × 0.75 mm^3^.

Prior to the MRS measurements, first- and second order *B*_0_ shimming of the MRS voxel of interest was performed using the fast, automatic shimming technique by mapping along projections (FASTESTMAP)^31^. Single-voxel MR spectra were measured using the stimulated echo acquisition mode (STEAM) sequence^32,33^ with ultra-short echo-time, TE = 4.6 ms; mixing time, TM = 28 ms; TR = 8200 ms; number of averages, 64; voxel-size, 20 mm (AP) × 20 mm (RL) × 20 mm (FH); RF pulse centred at 3.0 ppm, receive bandwidth, 6000 Hz; vector-size, 2048. The RF power was calibrated for each subject^34^. The sequence included water suppression (VAPOR) and outer-volume suppression (OVS) modules^33,35^. Additionally, two averages were measured without the water suppression RF pulses to record a water peak for eddy-current correction and quantification of the metabolite concentration in absolute units, based on the literature of tissue water concentrations^36^. The voxel positioning was performed by trained operators under the supervision of medical professionals.

### Data analysis

All spectra were pre-processed using the FID-A package^37^ implemented in MATLAB (Version 2022a). The pre-processing steps included automatic detection and removal of spectra strongly affected by motion^37^ and phase and frequency drift correction using spectral registration in the frequency domain^38^. Further spectral quantification was performed using LCModel Version 6.3-1R (http://s-provencher.com/lcmodel.shtml) with the water scaling and eddy current correction options enabled. Fitting was performed in the chemical shift range of 0.2–4.2 ppm. The metabolite basis set used in LCModel was generated with VeSPA^39^ for the STEAM sequence with ideal pulses, actual sequence timings, and previously published chemical shifts and J-coupling constants^40,41^. Nineteen metabolites were included in the basis set: Asp, *Myo*-Ins, Glu, Gln, GABA, alanine (Ala), ascorbate (Asc), creatine (Cr), glucose (Glc), glutathione (GSH), glycerophosphorylcholine (GPC), lactate (Lac), *N*-acetylaspartate (NAA), *N*-acetylaspartylglutamate (NAAG), phosphocreatine (PCr), phosphorylcholine (PCh), phosphorylethanolamine (PE), *scyllo*-inositol (*Scyllo*-Ins) and taurine (Tau). An experimentally acquired macromolecular (MM) spectrum measured using STEAM at 7 Tesla and similar experimental parameters was also included in the basis set^32^, which was obtained from the MM Consensus Data Collection repository (https://mrshub.org/datasets_mm). The LCModel output included estimated concentrations and Cramér-Rao lower bounds (CRLB), which are estimated standard deviations of the metabolite concentration in percentage. The latter was used to assure adequate quality and reliability. Only metabolite concentrations from individual subjects with a CRLB below 20% were included in the analysis. As the focus of the study is on *Myo*-Ins, Asp, Glu, Gln and GABA, further statistical analysis is restricted to these metabolites only.

Metabolite concentrations were quantified following the approach by Gasparovic et al.^36^, using the relaxation functions for the STEAM sequence^42^. The T1 and T2 relaxation time constants for water used in calculating the metabolite concentrations were obtained from previously published sources^43–46^. T1(GM) = 2132 ms, T1(WM) = 1220 ms, T1(CSF) = 4425 ms^45^; T2(GM) = 50 ms, T2(WM) = 55 ms, T2(CSF) = 141 ms^46^. Since the subjects are relatively young, relaxation changes due to age were not considered^47^. The relaxation time constants for neurochemicals were also not considered in calculating the metabolite concentrations due to their shorter T1 and longer T2 compared to water, resulting in a negligible effect as a result of our experimental setup (TE = 4.6 ms; TR = 8200 ms). To account for the partial volume of different tissues within the MRS voxel of interest, the MR structural images were segmented into grey matter (GM), white matter (WM), and cerebrospinal fluid (CSF) using FAST, whereas subcortical structures were segmented using FIRST tools implemented in FMRIB Software Library v6.0.3. Finally, the relative tissue volume within the MRS voxel of interest was estimated^48^. The average tissue composition of the PCC voxel was: FWHM: 0.026 ppm (SD: 0.004 ppm); S/N: 65.95 (SD: 5.75); CSF: 6.96% (SD: 2.87%); WM: 25.01% (SD: 2.87%) and GM: 68.03% (SD: 3.47%).

### Neurocognitive tests

Within seven days prior to or after the MRS measurement, participants underwent cognitive and psychopathological testing consisting of different tasks. Tasks were conducted on paper and on the computer and were preferably performed prior to MRI measurement in an undisturbed setting.

Testing included the Trail-Making Test A and B and the alertness and Go/No-go tasks from the TAP battery.

### Trail-making test

The Trail-Making Test (TMT) A/B is a well-established (1944) test that enables the quantification of visual search speed, processing, execution and overall attention^15^. Its popularity is due to its sensitivity to cognitive impairment^49^. In part A, participants are asked to connect circled numbers in ascending order as quickly as possible. The task examines the visual search and motor skills speed. In part B, alternating numbers and letters in consecutive order are connected (1-A-2-B and so on). This enables the examination of mental flexibility and complex processing speed. The total time needed to complete each TMT A and B is used in further analysis^15,50^.

### TAP battery

The TAP Battery is a computer-based group of tests created by Zimmermann and Fimm to assess attentional deficits. However, they are now also used to test and evaluate attentional functioning. Each subtest of the test battery assesses a different aspect of attention^17,18^. The subtests performed in this study were the following (TAP version 2.3.1 2017).

### Alertness task

The test measured participants’ visual reaction time to evaluate attention intensity, meaning the degree of focus and concentration as well as the speed and appropriacy of response. Tapping rapidity in response to the appearance of an “X” on the screen was utilised to evaluate the inherent level of arousal and wakefulness, referred to as intrinsic or tonic alertness. In the other part of the test, an acoustic warning stimulus was introduced before the presentation of the “X” with alternating time intervals of 300 ms and 700 ms. This allowed quantifying the so-called phasic arousal: a triggered state of temporarily heightened alertness^51,52^. The exercise duration was 6 min and consisted of four blocks of 20 trials, i.e., a total of 80 trials. The outcome variables used in this study are the median reaction time without signal (RT without signal median) with standard deviation (RT without signal SD) and the median reaction time with signal (RT with signal median) with standard deviation (RT with signal SD) as well as the phasic alertness value. Phasic alertness is an increase in response readiness for a brief duration triggered by an external stimulus^52^. The value is the difference of the RT median, with and without signal, divided by the total RT median^52,53^.

### Go/No-go task

The 1’ of 2’ stimuli version task was performed. Here, participants briefly saw a randomly alternating “X” and “+” appearing on the screen and had to quickly tap when the “X” appeared. The task consisted of 60 trials (24 “X” and 36 “+”) in total, enabling measurement of speed and response control as well as the reaction suppression to a stimulus—all of which are factors in selective attention and focused attention. The outcome scores used were the mean of the reaction time for correct answers and the number of mistakes.

### Dropouts and missing data/descriptive statistics

Spectroscopy data from two participants could not be reliably measured. TAP data of one participant was missing. Cases with missing values for any variable were excluded from the analysis, resulting in a total of 43 complete data sets.

### Statistical analysis

Multiple linear regression was performed for each cognitive test and metabolite with gender and age as covariates. Bootstrapping with a simple replacement was additionally performed to achieve more robust results.

Data analysis was performed using SPSS Version 29.0.0.0 (241). (https://www.ibm.com/products/spss-statistics) Multiple linear regression analysis was performed individually for each of the nine cognitive test results (dependent variable) and the concentration of the five metabolites (independent variable). This resulted in a total of 45 regressions. Age and gender were included as covariates of no interest in all linear regression analyses to mitigate their influence and to prevent potential confounding of the observed effects. Predictors were entered into the model simultaneously with the forced entry method. Coefficients with a *p*-value < 0.05 were considered significant.

Assumptions were tested. Multicollinearity among predictors was assessed using the VIF (Variance Inflation Factor) values, with the minimum cut-off set at one and the maximum at 10. Cook’s distances were calculated to assess the influence of individual data points on the model. Notably, all Cook’s values were insignificant (Cook < 1), indicating no influential outliers. The normality of the residuals was examined with the Shapiro–Wilk test, and scatterplots were used to visually inspect their homoscedasticity. Some of the results violated the latter assumptions; however, this issue was mitigated using bootstrapping^54^.

### Bootstrapping

Bootstrapping was performed using simple random sampling with replacement (1000 number of samples and confidence interval: 95%)^55^. By generating multiple bootstrap samples and examining the variability of the coefficients across these samples, the representation of the population was effectively captured. The primary objective of bootstrapping was to mitigate the accumulation of alpha errors in the context of multiple testing and provide more robust and reliable results.

## Results

### Neurocognitive tests

The results of the neurocognitive tests can be seen in Table 1.

**Table 1:** Descriptive statistics of the neurocognitive tests.

Neurocognitive test	Mean and SD
TMT A (n = 46)	23.52 s ± 8.13 s
TMT B (n = 46)	51.07 s ± 14.75 s
Alertness without signal median (n = 45)	225.84 ms ± 24.38 ms
Alertness without signal SD (n = 45)	28.82 ms ± 11.21 ms
Alertness with signal median (n = 45)	224.16 ms ± 22.45 ms
Alertness with signal SD (n = 45)	27.47 ms ± 11.36 ms
Phasic alertness (n = 45)	0.006 ± 0.065
Go/No-go mean (n = 45)	379.87 ms ± 47.09 ms
Go/No-go mistakes (n = 45)	0.73 ± 0.96

TMT A = Trail Making Test A; TMT B = Trail Making Test B; Alertness without signal median = median reaction time of alertness task without signal; Alertness without signal SD = standard deviation of alertness without signal; Alertness with signal median = median reaction time of alertness task with signal; Alertness with signal SD = standard deviation of alertness with signal; Go/No-go mean = Mean of the reaction time in Go/No-go task; Go/No-go mistakes = Number of mistakes in the Go/No-go task.

### MRS

**Figure 1:**
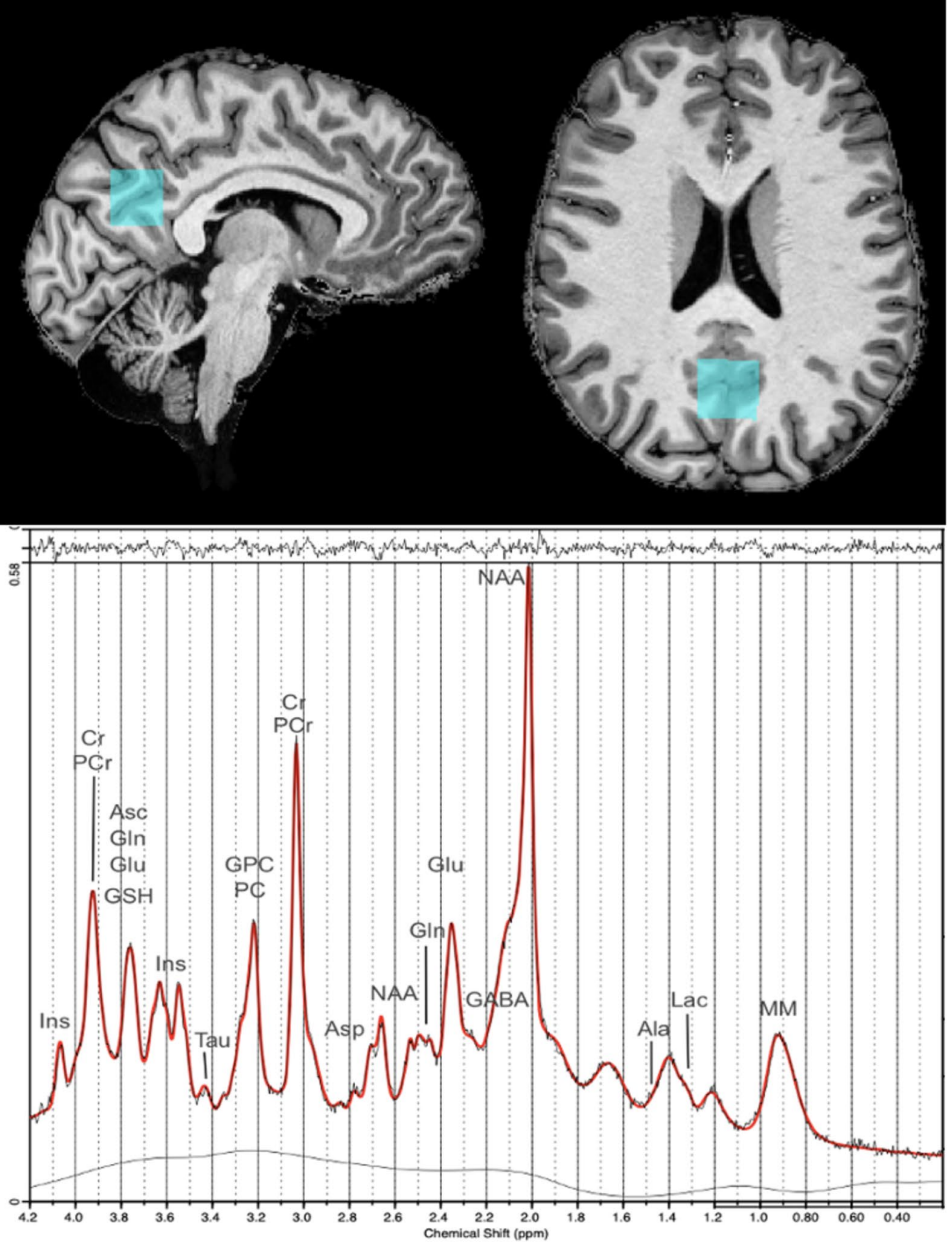
Example of an LCModel outcome. Top: a representative in vivo positioning of voxel of interest. Bottom: a H1 MRS spectrum from a 27-year-old woman.

The concentration of Asp, Glu, Gln, GABA and *Myo*-Ins in the PCC were reliably detected in 44 participants. The location of the MRS voxel of interest in the PCC and the corresponding experimental spectrum for an exemplary participant are given in Fig. 1.

**Figure 2:**
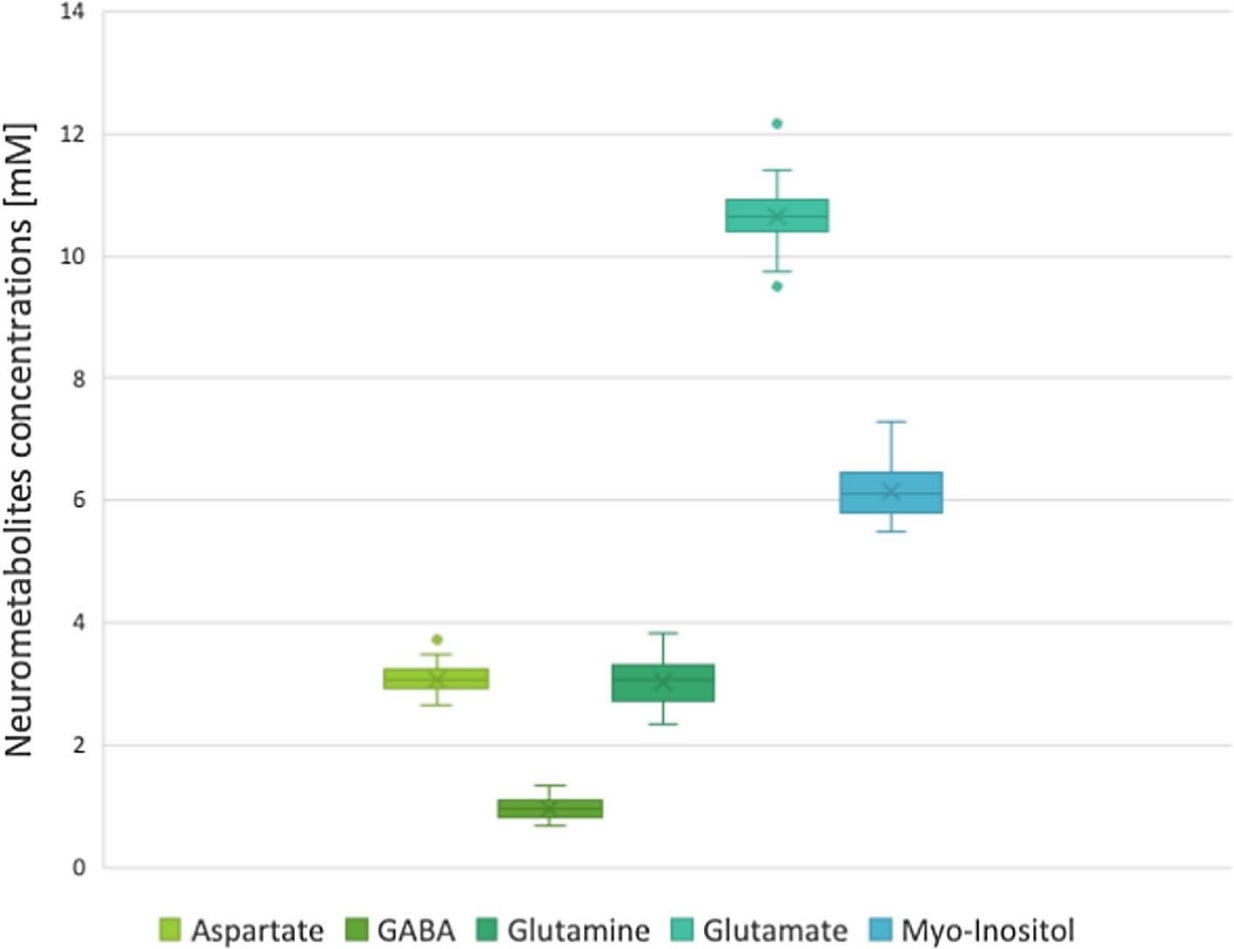
Box plots of concentrations of measured metabolites in mM. With Glutamate having the highest concentrations and GABA the lowest.

Figure 2 shows the mean and standard deviation of Asp, GABA, Gln, Glu and *Myo*-Ins concentrations in the PCC. The average FWHM (full width at half-maximum, an estimate of the linewidth in the in-vivo MR spectra), across the subjects as reported by LCModel was 0.026 ppm ± 0.004 ppm. The average signal-to-noise ratio (SNR, defined as the ratio of the maximum in the spectrum-minus baseline over the analysis window to twice the root mean square residuals) of the water-suppressed MR spectra across the subjects as reported by LCMOdel was 65.95 ± 5.75. The average tissue composition across the subjects in the PCC voxel was, CSF: 6.96 ± 2.87%; WM: 25.01 ± 2.87%; GM: 68.03 ± 3.47%. The average CRLB across the subjects of the included metabolites were, Ins: 2.04 ± 0.21%; Asp: 8.75 ± 0.94%; Glu 1.5 ± 0.51%; Gln: 4.12 ± 0.65%; GABA: 12.5 ± 2.53%.

### Predictions

The findings of the multiple linear regression analyses demonstrated several consistent and robust results, which persisted even after performing bootstrapping (BS). The results of the coefficient (CE) table for the metabolites can be seen in Table 2.

**Table 2:** Multiple linear regression table of n = 43 participants with cognitive tests as dependent variables.

	Glutamate		Glutamine		GABA		*Myo*-Inositol		Aspartate	
TMT A	B	− 3.565	B	− 5.002	B	− 1.467	B	− 8.837	B	− 8.600
	SE B	2.356	SE B	3.805	SE B	8.701	SE B	3.044	SE B	5.716
	β	− 0.238	β	− 0.219	β	− 0.028	β	− 0.431	β	− 0.229
	p (CE)	0.138	p (CE)	0.196	p (CE)	0.867	p (CE)	**0.006**	p (CE)	0.140
	p (BS)	0.226	p (BS)	0.336	p (BS)	0.832	p (BS)	**0.008**	p (BS)	0.120
	Bootstrap CI	(− 10.298) − (1.706)	Bootstrap CI	(− 14.094) − (4.284)	Bootstrap CI	(− 17.335) − (12.883)	Bootstrap CI	(− 14.994) − (− 3.468)	Bootstrap CI	(− 20.610) − (0.853)
TMT B	B	− 6.410	B	− 3.717	B	− 15.285	B	− 15.207	B	− 8.998
	SE B	4.287	SE B	7.043	SE B	16.447	SE B	5.596	SE B	10.591
	β	− 0.237	β	− 0.090	β	− 0.155	β	− 0.411	β	− 0.133
	p (CE)	0.143	p (CE)	0.601	p (CE)	0.358	p (CE)	**0.010**	p (CE)	0.401
	p (BS)	0.224	p (BS)	0.590	p (BS)	0.296	p (BS)	**0.027**	p (BS)	0.310
	Bootstrap CI	(− 17.782) − (2.461)	Bootstrap CI	(− 17.557) − (9.887)	Bootstrap CI	(− 47.505) − (15.557)	Bootstrap CI	(− 28.408) − (− 3.212)	Bootstrap CI	(− 28.641) − (7.713)
*Alertness without signal Median*	B	− 8.267	B	− 3.794	B	2.268	B	− 1.764	B	− 31.413
	SE B	7.109	SE B	11.713	SE B	27.334	SE B	9.873	SE B	16.578
	β	− 0.181	β	− 0.055	β	0.014	β	− 0.029	β	− 0.281
	p (CE)	0.252	p (CE)	0.748	p (CE)	0.934	p (CE)	0.859	p	0.066
	p (BS)	0.282	p (BS)	0.688	p (BS)	0.936	p (BS)	0.882	Bootstrap p	0.070
	Bootstrap CI	(− 22.994) − (8.135)	Bootstrap CI	(− 25.461) − (15.289)	Bootstrap CI	(− 47.127) − (47.575)	Bootstrap CI	(− 20.740) − (20.730)	Boostrap CI	(− 64.486) − (3.301)
*Alertness without Signal SD*	B	− 0.934	B	− 6.460	B	19.719	B	2.347	B	− 17.996
	SE B	3.466	SE B	5.530	SE B	12.699	SE B	4.723	SE B	7.795
	β	− 0.044	β	− 0.205	β	0.257	β	0.083	β	− 0.349
	p (CE)	0.789	p (CE)	0.250	p (CE)	0.129	p (CE)	0.622	p (CE)	**0.026**
	p (BS)	0.816	p (BS)	0.319	p (BS)	0.199	p (BS)	0.540	p (BS)	0.096
	Bootstrap CI	(− 8.366) − (6.477)	Bootstrap CI	(− 18.639) − (4.730)	Bootstrap CI	(− 9.258) − (45.402)	Bootstrap CI	(− 5.799) − (10.024)	Bootstrap CI	(− 39.912) − (0.604)
*Alertness with Signal Median*	B	− 0.008	B	0.221	B	4.990	B	4.808	B	− 16.867
	SE B	5.197	SE B	8.609	SE B	19.713	SE B	7.097	SE B	12.289
	β	0.000	β	0.005	β	0.043	β	0.111	β	− 0.215
	p (CE)	0.999	p (CE)	0.980	p (CE)	0.802	p (CE)	0.502	p (CE)	0.178
	p (BS)	1.000	p (BS)	0.978	p (BS)	0.757	p (BS)	0.523	p (BS)	0.193
	Bootstrap CI	(− 10.321) − (11.148)	Bootstrap CI	(− 16.648) − (17.002)	Bootstrap CI	(− 27.895) − (40.711)	Bootstrap CI	(− 8.145) − (21.535)	Bootstrap CI	(− 43.818) − (5.518)
*Alertness with signal SD*	B	− 3.814	B	− 1.158	B	12.113	B	1.710	B	− 7.175
	SE B	3.407	SE B	5.611	SE B	12.865	SE B	4.719	SE B	8.212
	β	− 0.179	β	− 0.036	β	0.157	β	0.060	β	− 0.138
	p (CE)	0.270	p (CE)	0.838	p (CE)	0.352	p (CE)	0.719	p (CE)	0.388
	p (BS)	0.242	p (BS)	0.855	p (BS)	0.145	p (BS)	0.658	p (BS)	0.421
	Bootstrap CI	(− 10.123) − (3.002)	Bootstrap CI	(− 13.608) − (11.409)	Bootstrap CI	(− 6.577) − (27.184)	Bootstrap CI	(− 5.377) − (9.011)	Bootstrap CI	(− 24.783) − (9.133)
*Phasic Alertness*	B	− 0.033	B	− 0.052	B	0.000	B	− 0.014	B	− 0.026
	SE B	0.019	SE B	0.032	SE B	0.076	SE B	0.027	SE B	0.048
	β	− 0.271	β	− 0.284	β	0.001	β	− 0.085	β	− 0.087
	p (CE)	0.096	p (CE)	0.106	p (CE)	0.995	p (CE)	0.615	p (CE)	0.589
	p (BS)	0.066	p (BS)	0.077	p (BS)	0.997	p (BS)	0.562	p (BS)	0.626
	Bootstrap CI	(− 0.073) − (0.004)	Bootstrap CI	(− 0.106) − (0.003)	Bootstrap CI	(− 0.153) − (0.131)	Bootstrap CI	(− 0.054) − (0.042)	Bootstrap CI	(− 0.109) − (0.108)
*Go/No-go Mean*	B	1.681	B	− 10.977	B	35.879	B	11.868	B	− 72.405
	SE B	14.224	SE B	23.006	SE B	53.229	SE B	19.337	SE B	32.050
	β	0.019	β	− 0.083	β	0.113	β	0.100	β	− 0.337
	p (CE)	0.907	p (CE)	0.636	p (CE)	0.504	p (CE)	0.543	p (CE)	**0.030**
	p (BS)	0.924	p (BS)	0.608	p (BS)	0.553	p (BS)	0.548	p (BS)	0.097
	Bootstrap CI	(− 32.058) − (34.624)	Bootstrap CI	(− 51.117) − (28.221)	Bootstrap CI	(− 78.132) − (145.463)	Bootstrap CI	(− 34.618) − (51.005)	Bootstrap CI	(− 157.084) − (0.542)
*Go/No-go Mistakes*	B	− 0.143	B	0.176	B	0.032	B	− 0.177	B	1.808
	SE B	0.285	SE B	0.462	SE B	1.077	SE B	0.389	SE B	0.620
	β	− 0.082	β	0.068	β	0.005	β	0.389	β	0.426
	p (CE)	0.619	p (CE)	0.705	p (CE)	0.976	p (CE)	0.653	p (CE)	**0.006**
	p (BS)	0.604	p (BS)	0.721	p (BS)	0.982	p (BS)	0.615	p (BS)	**0.008**
	Bootstrap CI	(− 0.733) − (0.482)	Bootstrap CI	(− 0.665) − (1.166)	Bootstrap CI	(− 2.190) − (2.638)	Bootstrap CI	(− 0.948) − (0.617)	Bootstrap CI	(0.525) − (2.998)

The independent variables are each individual metabolite, age and gender. The values for the covariates age and gender are not represented in this table because they were of no interest. Cognitive tests: TMT A = Trail Making Test A; TMT B = Trail Making Test B; Alertness without signal median = median reaction time of alertness task without signal; Alertness without signal SD = standard deviation of alertness without signal; Alertness with signal median = median reaction time of alertness task with signal; Alertness with signal SD = standard deviation of alertness with signal; Go/No-go mean = Mean of the reaction time in the Go/No-go task; Go/No-go mistakes = Number of Mistakes in the Go/No-go task. Metabolites: Asp = aspartate; *Myo*-Ins = *myo*-inositol; Glu = glutamate; Gln = glutamine; GABA = gamma aminobutyric acid. Covariate table with values of metabolites: B = unstandardised beta; SE B = standard error beta; β = beta, standardized coefficient; p(CE) = *p* value of metabolite coefficient. Values for bootstrapping: p(BS) = p after the bootstrapping; BS CI = lower and upper 95% confidence interval value of bootstrapping. * = *p* < 0.05; ** = *p* < 0.01.

Significant values are in bold.

**Figure 3:**
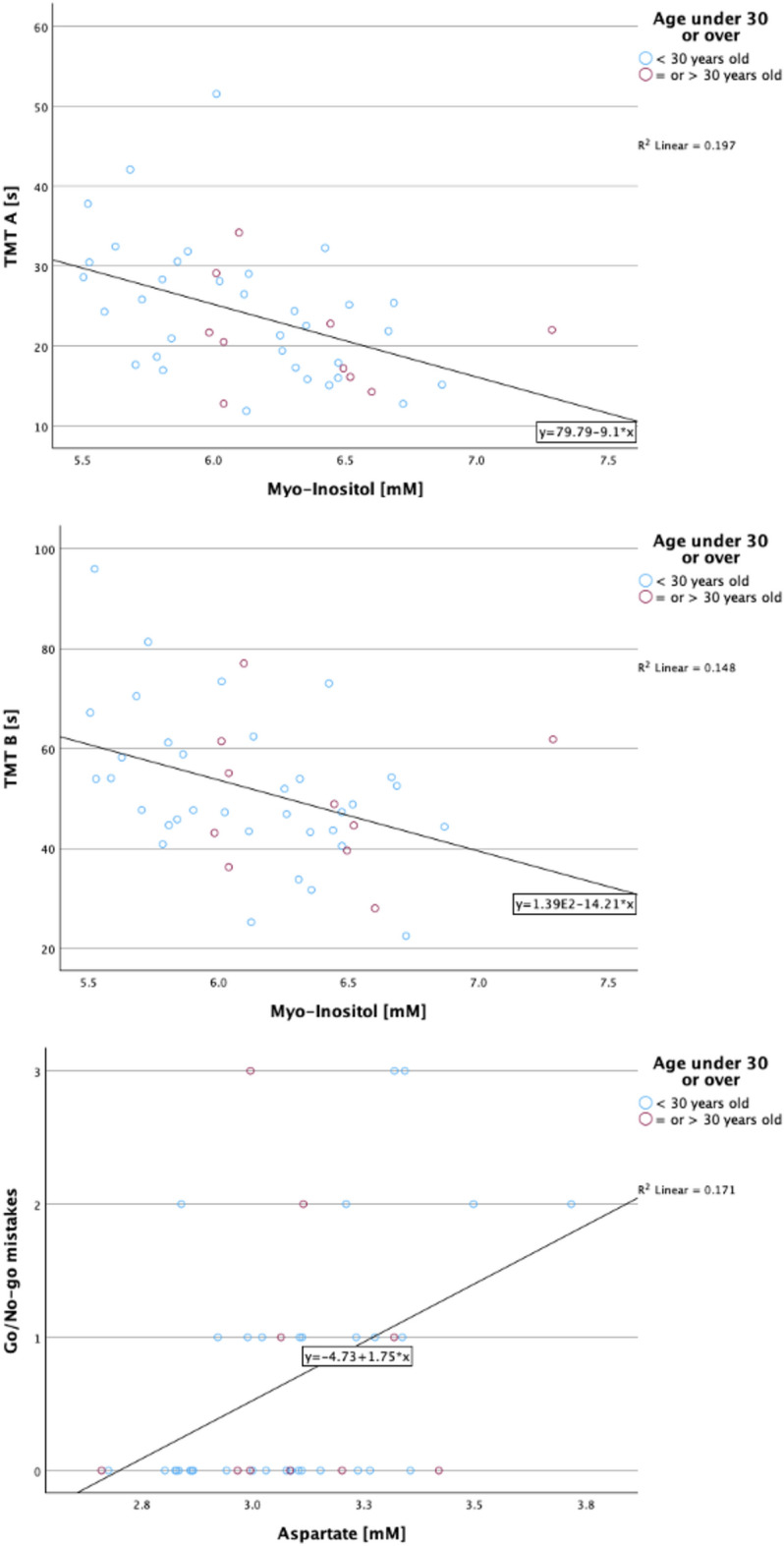
Scatterplots illustrating the relationship between cognitive performance and metabolite concentrations that were significant. Top: TMT A scores versus *myo*-inositol levels, Middle: TMT B scores versus *myo*-inositol levels, Bottom: Go/No-go mistakes versus aspartate levels. Each plot includes a regression line indicating the trend of the data.

The influence of *Myo*-Ins on both TMT A and B was significant (*p*(*BS*) = 0.008 and *p*(*BS*) = 0.027), as was the influence of Asp on Go/No-go mistakes (*p*(*BS*) = 0.008). Fig. 3 shows scatterplots representing the relationship between *Myo*-Ins and TMT A and B, as well as Asp and Go/No-go mistakes.

A trend towards regression was observed between Asp and the mean Go/No-go value (pre-BS: *p*(CE) = 0.030, post-BS: *p* (BS) = 0.097), as well as with alertness without signal SD (pre-BS: *p*(CE) = 0.026, post-BS: *p*(BS) = 0.096). However, it is important to note that these associations did not retain statistical significance (*p* < 0.05) following bootstrapping. Additionally, Asp had a marginal effect on alertness without signal median (pre-BS: *p*(CE) = 0.066, post-BS: *p*(BS) = 0.070). No significant (*p* < 0.05) relationships were found between Glu, Gln, GABA, and cognitive performance. The complete coefficient tables of the significant results can be found on supplementary Fig. S2–S4.

## Discussion

Using UHF MRS, concentrations of Glu, Gln, GABA, Asp and *Myo*-Ins in the PCC were precisely obtained and examined to determine if these metabolites could predict the outcome in attentional tasks, such as alertness and executive function. This was found to be the case for *Myo*-Ins and Asp, thereby improving our understanding of the role of certain metabolites in cognitive tasks involving attention.

The first notable result was the relationship between *Myo*-Ins and the performance in the TMT tests. Higher levels of *Myo*-Ins were associated with better results in TMT A and B. This indicates that the concentration of *Myo*-Ins in the PCC can predict visual search speed, processing speed and mental flexibility. Ergo, *Myo*-Ins influences both executive function and the orienting part of attention to a degree^14^. Interestingly, in other branches of research, such as nutrition, recent studies have already reported positive effects on processing speed when supplementing with pills containing inositol-stabilised arginine silicate (ASI)^51,56^. Considering that when *Myo*-Ins gets ingested it lands in the brain, among other things probably because of his property as a brain osmolyte^57,58^. Moreover, several studies researching ASI showed a better performance in the TMT, supporting the hypothesis that *Myo*-Ins may indeed have an influence on attention^59^,^60^. A possible explanation for the influence of *Myo*-Ins on cognition is its involvement in the arginine metabolism^61^. It has been observed that *Myo*-Ins reduces arginase activity, which in turn leads to increased amount of arginine. Arginine is a semi-essential amino acid that is beneficial in functions such as cell proliferation^62^ and immune response^63^, as well as in stress suppression and regulation^64^. Not only is arginine a precursor of nitric oxide, glutamate, polyamines, proline, creatinine and agmatine, but its availability also affects gene expression^65–67^. The positive effect of arginine on brain metabolism, and thus cognition, can be attributed to the production of nitric oxide, a free radical known to induce vasodilation^68^. As a result, enhanced cerebral blood flow facilitates improved oxygen and glucose delivery to brain regions such as the PCC, thus improving its function and the modulation of activation and deactivation.

However, in contrast to our findings, elevated *Myo*-Ins concentrations have been negatively correlated with cognitive performance in the CAMCOG examination, a test for evaluating cognitive impairment in individuals with Down Syndrome (DS)^69^. That being said, the study used *Myo*-Ins concentrations measured in the hippocampus rather than the PCC, and the correlation was observed in individuals with DS. These findings could indicate that *Myo*-Ins has distinct functions in different regions of the brain and raise the possibility that altered gene expression contributes to differences in *Myo*-Ins function. Despite these apparently contradictory findings, *Myo*-Ins represents a promising metabolite that could be harnessed to strengthen cognitive performance, and therefore, further research is needed to understand its role fully. Lastly, we would like to stress the discrepancy of our findings with the commonly accepted link between elevated *Myo*-Ins levels and neurodegeneration^70^, as our MRS observations suggest a pathomechanism for *Myo*-Ins that appears unrelated to neurodegeneration.

The second finding of our study was a significant (*p* < *0.05*) association between Asp concentration and the number of mistakes made in the Go/No-go task, suggesting that higher Asp levels result in a diminished capacity for reaction suppression. Furthermore, the observed trends (see Table 2) indicate that Asp also possibly affects decision-making speed, as characterised by outcomes from the Go/No-go mean, and enhances reaction stability, as indicated by reduced variability considering alertness without signal SD. These results demonstrate the influence of Asp on the executive system and possibly on the alerting system of attention, although this is just a trend^14^.

As Asp is only detectable with UHF MRS scanners, literature on its function is limited.

However, it has been established that Asp acts as a major excitatory neurotransmitter which activates several receptors, including the N-methyl-D-aspartate (NMDA) receptor^21^. More recent pharmacological studies further support this notion by demonstrating that D-Asp acts as an agonist on NMDA, mGlu5 and AMPA/kainate receptors, exciting neurons, as well as inducing Glu release in specific brain regions^71,72^. Conversely, blocking hippocampal AMPA receptors in rats through Asp has also been observed, demonstrating its involvement in synaptic transmission^73^.

One possible explanation for the results obtained in this study is that as an excitatory metabolite, higher Asp levels could lead to reduced DMN deactivation in a similar way to Glu^74^. This reduced deactivation possibly results in impaired attention and the decreased ability to suppress reactions.

Asp is an amino acid that is a vital component of the mammalian metabolism. As an amino acid, it can be differentiated in its two chiral forms L-Asp and D-Asp, L-Asp being the format mainly used to build proteins. The current state of knowledge indicates that D-Asp plays a significant role in the endocrinological cycle and that it is abundant in the brains of mammalian embryos. Its decrease following development from the embryonic state is due to the D-Asp oxidase (DDO) that catabolises it. To better understand the role of D-Asp, De Rosa et al. generated genetically modified mice with overexpressed DDO that led to a strong decrease of D-Asp in the brain^75^. The study showed that the intervention did not lead to any alteration in the composition of the glutamatergic cycle, but a decrease in the number of GABAergic parvalbumin interneurons, which are described to play a role in cognitive-behavioural deficits in psychiatric conditions, was apparent^76^. They also found improved spatial memory and object recognition in the mice with less D-Asp, indicating an influence on cognition^75^. Another study conducted on mice administered with D-Asp also demonstrated an improvement in memory consolidation and an upregulation of hippocampal NMDA-receptors, leading to long-term potentiation (LTP) onset and to the stabilisation of LTDs traces^77^. Facilitated late-phase LTP in the prefrontal cortex has also been indicated in a study involving humans with chronically elevated D-Asp^78^. It should be noted, that the role of aspartate as an neurotransmitter for the NMDA receptors in the hippocampus, which are involved in the induction of LTPs, is disputed^79^. Given these findings and considering that our study, as far as we are aware, is the first MRS investigation proposing a link between Asp levels in the PCC and attentional performance, there is a need for further research to explore the influence of Asp in various brain regions, including the PCC.

The function of GABA in cognition is a frequently explored topic in neuroscience, and the role of GABAergic interneurons in generating gamma waves, which are associated with cognitive processes, including attention and memory, is well discussed^80,81^. In order for effective engagement in tasks involving externally directed attention to occur, deactivation in the PCC is required^2,82^ and GABA has been demonstrated to enhance this deactivation, suggesting its potential function in the performance of attentional tasks^25^. This aligns with findings in ageing populations, which show a correlation between declining GABA levels and deterioration of cognitive function^83,84^. There was, however, a distinction between anterior cerebral midline and posterior cerebral midline structures, whereby only the GABA concentration in the former appears to be a predictor for cognitive performance^84^. This is consistent with our findings, according to which the GABA concentration in the PCC (posterior brain part) does not seem to influence attention. A further 3T MRS study measuring patients with relapsing multiple sclerosis found that lower GABA concentrations in the PCC correlate with decreased executive performance in the TMT. However, this study did not observe any significant (*p* < *0.05*) association between GABA levels and any cognitive test in their control group^85^. This observation also agrees with our findings obtained from healthy subjects. Noteworthy is that most spectroscopic studies examining the influence of GABA on cognition have identified brain regions such as the frontal lobe, the anterior cingulate cortex and the hippocampus as being the areas that demonstrate significant associations with cognition, whereas the PCC is considered to be less involved^86,87^.

DMN activation and deactivation is not only influenced by GABA but also by Glu, which, in larger amounts, causes decreased deactivation^74^. Insufficient deactivation of the DNM can lead to impaired externally directed focus and consecutively cognition^7^. Previous research has demonstrated the involvement of Glu in cognition and particularly in memory^86,88, 89^. Decreased Glu levels in the PCC, associated with neuronal loss in conditions such as Alzheimer’s disease or mild cognitive impairment, seem to be one of the reasons for the memory dysfunction and cognitive decline associated with these conditions^86,90^. Recently, Matthews et al. demonstrated how riluzole, a neuroprotective drug, supported higher Glu levels in the PCC of patients with Alzheimer’s disease and observed a correlation between higher PCC Glu levels and better cognitive performance in the Mini Mental State Examination and the Alzheimer’s Disease Assessment Scale-Cognitive Subscale^91^. Although our study, which focuses on attentional performance, does not suggest a relationship between Glu or Gln concentrations in the PCC and cognitive functions, it would be a valuable area for further investigation.

The limitations of this study include the small sample size and the fact that most participants were young and from a similar social demographic, potentially leading to a selection bias.

In our analysis, although scyllo-inositol and glucose were included in the basis set, the high CRLB values (> 50%) outputted by LCModel indicate significant uncertainty. Therefore, we have refrained from presenting data for these metabolites. Future studies with enhanced detection capabilities or alternative analysis methods may be required to reliably quantify these metabolites.

In conclusion, the present 7T MRS neuropsychiatric study investigated the influence of key metabolites in the PCC on attention. The results indicate that the anti-oxidative sugar *Myo*-Ins has a positive effect on visual search, processing speed and mental flexibility, while the excitatory amino acid Asp appears to be associated with poorer suppression control. No influence on attention was observed for Glu, Gln and GABA. These findings provide novel insights into the role of *Myo*-Ins and Asp in the brain of healthy individuals. However, further research is required to elucidate the mechanisms through which *Myo*-Ins and Asp modulate cognitive processes.

### Supplementary Information

Supplementary Figures.

## Data Availability

The data used can be accessed by contacting the principal investigator, Prof. Dr. Neuner.
