# Supplementary material for: Predicting performance in attention by measuring key metabolites in the PCC with 7T MRS
Source: Sci Rep. 2024 Jul 24;14:17099. doi: 10.1038/s41598-024-67866-1 (PMC11269673; doi:10.1038/s41598-024-67866-1)
Supplement: Supplementary file 1 — Supplementary Figures. [file 41598_2024_67866_MOESM1_ESM.docx]

**Predicting performance in attention by measuring key metabolites in the PCC with 7T MRS**

Collée M^i,ii^, Rajkumar R^i,ii,iii^, Farrher E^ii^, Hagen J^i,ii^, Ramkiran S^i,ii^, Schnellbächer GJ^i,ii^, Khudeish, N^i,ii^, Shah NJ^i,ii,iii,iv,v^, Veselinović T ^i,ii,iii^, Neuner I ^i,ii,iii^

Supplementary Materials

***
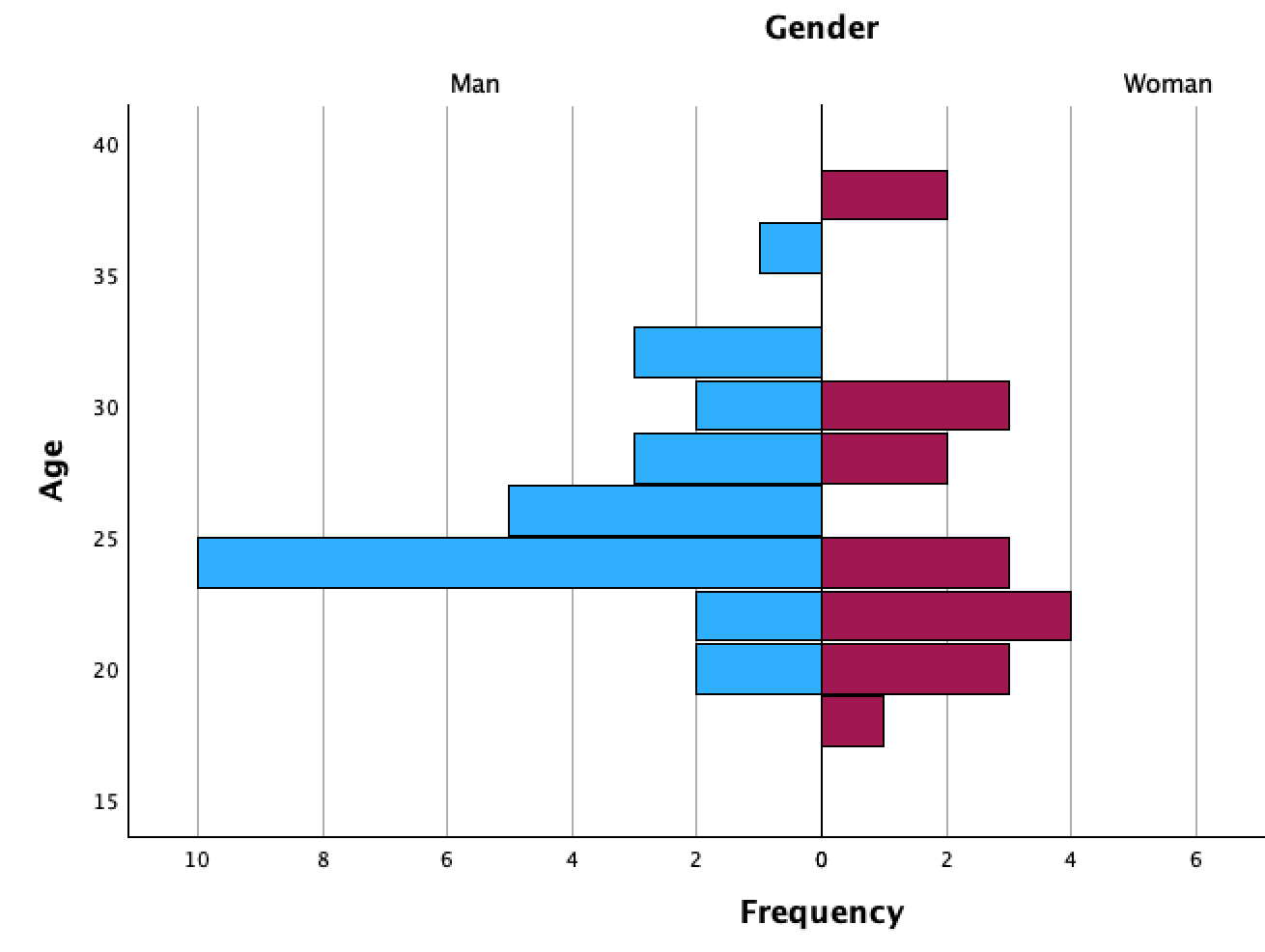
***

Supplementary Fig. S1: Population pyramid of participants. Men are displayed on the left, women on the right.


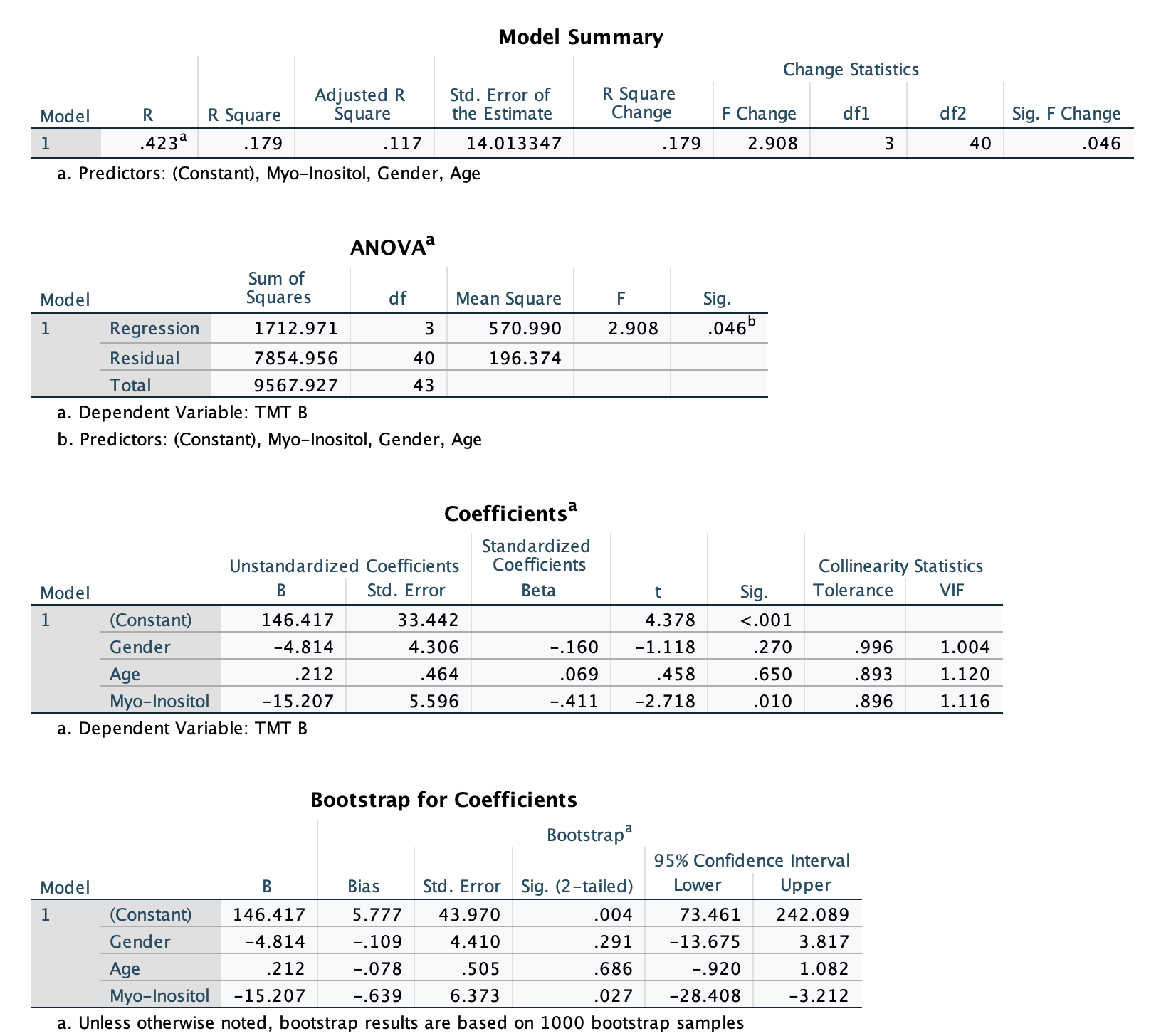


Supplementary Fig. S2: SPSS Output of multiple regression with TMT B as dependent variable and myo-inositol, age and gender as predictors. The VIF values close to 1 suggest that the predictor variables exhibit minimal multicollinearity, indicating that there is no substantial linear relationship between them.


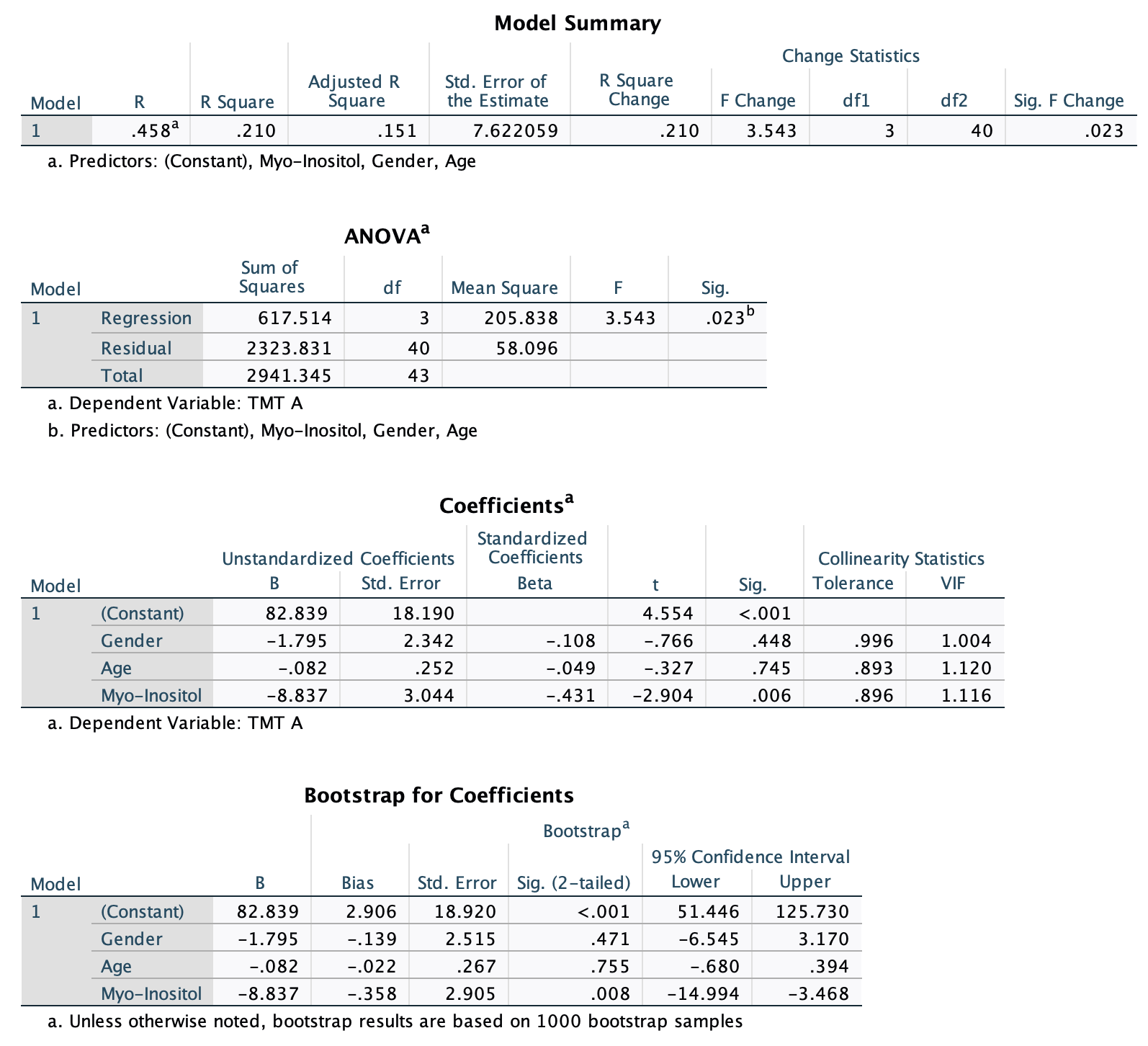


Supplementary Fig. S3: SPSS Output of multiple regression with TMT A as dependent variable and myo-inositol, age and gender as predictors. The VIF values close to 1 suggest that the predictor variables exhibit minimal multicollinearity, indicating that there is no substantial linear relationship between them.


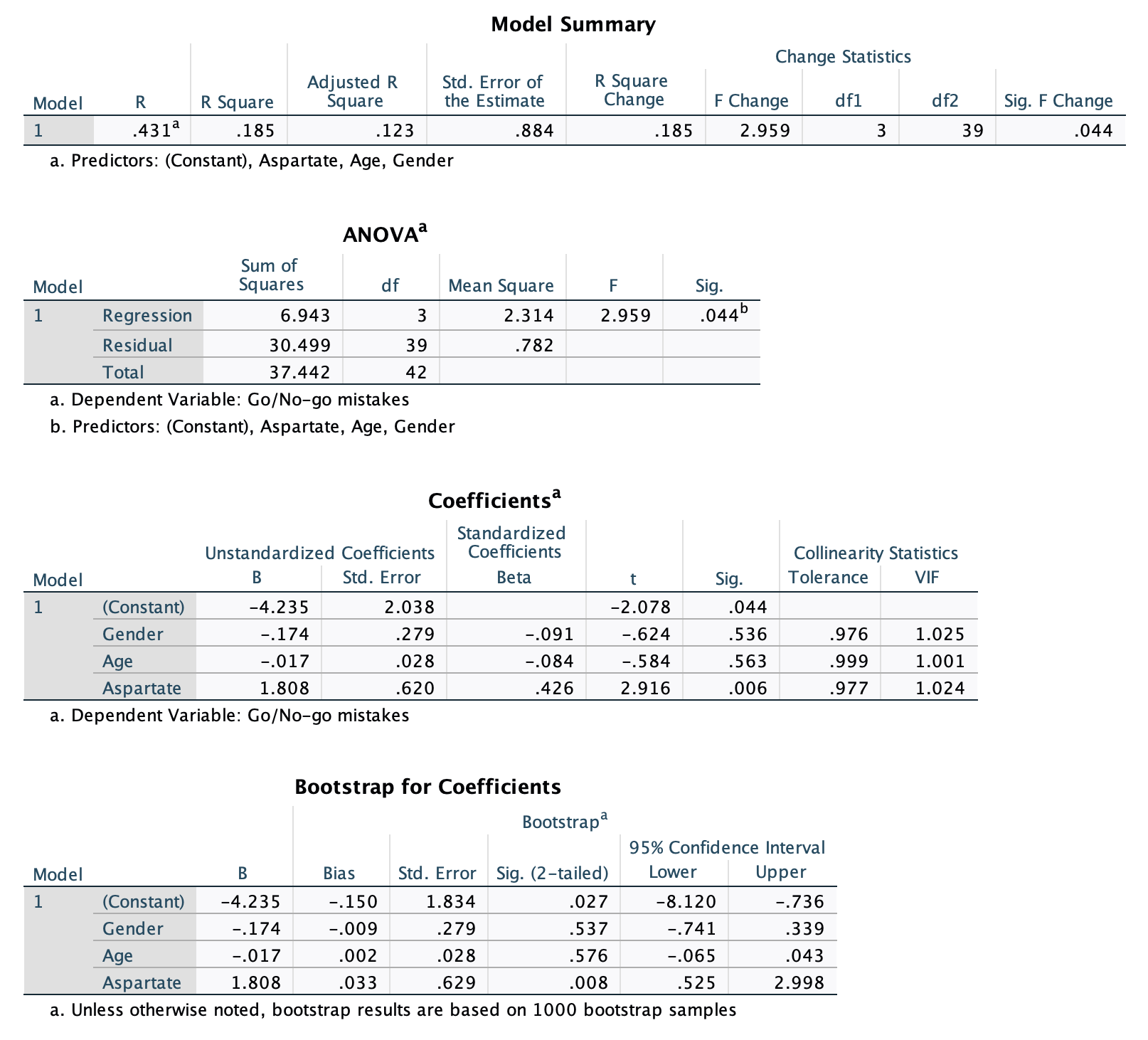


Supplementary Fig. S4: SPSS Output of multiple regression with Go/Nogo Mistakes as dependent variable and aspartate, age and gender as predictors. The VIF values close to 1 suggest that the predictor variables exhibit minimal multicollinearity, indicating that there is no substantial linear relationship between them.
